# Interlaboratory Study to Minimize Wavelength Calibration Uncertainty Due to Peak Fitting of Reference Material Spectra in Raman Spectroscopy

**DOI:** 10.1177/00037028251330654

**Published:** 2025-04-24

**Authors:** Dirk Lellinger, James Thomson, Nicolas Coca-Lopez, Afroditi Ntziouni, Nikolaos Nikoloudakis, María Fernández-Álvarez, Nina Jeliazkova, Miguel A. Bañares, Raquel Portela, Enrique Lozano Diz

**Affiliations:** 1 Fraunhofer LBF, Darmstadt, Germany; 2 ELODIZ Ltd., High Wycombe, UK; 3 Instituto de Catalisis y Petroleoquimica (ICP), CSIC, Madrid, Spain; 4 NTUA, Athens, Greece; 5 Instituto de Ceramica y Vidrio (ICV), CSIC, Madrid, Spain; 6 Ideaconsult Ltd., Bulgaria

**Keywords:** Peak shape, signal-to-noise ratio, S/N, neon, silicon, polystyrene, calcite, instrument verification, standard

## Abstract

Raman spectroscopy is a powerful characterization technique with increasing applications that would greatly benefit from data harmonization. Several standards deal with calibration in Raman spectroscopy, but no detailed procedure covers the complete calibration of an instrument, including both spectral axes, from reference material spectra generation to data processing. Moreover, the type of reference materials, the quality of the recorded spectra and the choice of the fitting functions are critical for obtaining precise and reliable reference data for calibration. This report describes the challenges and importance of peak fitting for Raman signal calibration based on an interlaboratory study with 10 different instruments. Spectra of neon emission, silicon, calcite, and polystyrene were fitted using common peak shapes, observing that Gaussian, Pearson IV, Voigt, and Voigt shapes are preferred for these materials, respectively. An analysis of the effect on the fitting of the signal-to-noise ratio (S/N) recommends a minimum value of 100 for a Raman peak if it should be used to calibrate a Raman instrument. Some factors that might affect the peak shape of the Raman signal, such as the physical and chemical properties of the sample, the nature of the electronic transitions, the instrument response and the spectral resolution are discussed. The results highlight the role of peak fitting analysis in improving the quality and reliability of Raman spectra calibration and, thus, enhancing data transfer and comparability, especially for handheld and portable Raman analyzers, as well as applications based on quantification, multivariate data analysis, and other complex processing steps.

## Graphical abstract

**Graphical abstract:**
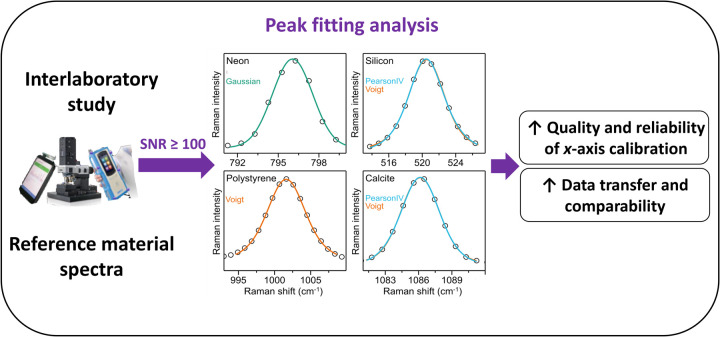
This is a visual representation of the abstract.

## Introduction

Several guidelines, publications, and standards provide instructions for calibration in Raman spectroscopy,^1–4^ but there is a need for a unified and comprehensive protocol that covers the full instrument calibration process, from reference materials spectra generation to details on how to manage data processing, for both axes of the spectrum. When calibrating a Raman instrument, one of the major challenges is getting precise and reliable reference information; this, in turn, depends on the quality of the reference materials, how they are measured, and how the data are processed.^5,6^ In this regard, it is important to use the most suitable fitting function for each step of the calibration process to extract the best estimates of the position, intensity, and width of individual emission and Raman peaks.^7^ The observed Raman peaks are a convolution of the laser emission line shape, the sample’s Raman scattering, and the instrument response.^8^ Because of the nature of the electronic transitions of the materials used in the calibration of Raman instruments and the way these signals are read, the peaks of the reference material spectra are expected to fall under a limited number of line shapes: solid samples generate a more Gaussian-like peak, gases more often a Lorentzian-shaped peak, and liquids a combination of both (Voigt or Gaussian–Lorentzian peaks).^9^ However, there are physicochemical factors like crystallinity, state of the material, presence of doping elements or isotopic forms, temperature, resonance effects, and many others that can lead to peak broadening, overlapping or asymmetry, hence altering this general statement. These changes can have a significant effect on the resulting calibration of the instrument if not correctly accounted for. Asymmetric Raman peaks, for example, are typically best fit using Pearson IV function.^10^ The overlapping of peaks due to low spectral resolution and/or the use of reference materials with concurrent similar peaks (e.g*.*, from different polymorphic forms, crystalline phases, or impurities in the sample) is one of the several factors that can alter the peak shape of a Raman signal used for calibration purposes.^7^ Raman spectroscopy is nevertheless a well-established technology, as the readouts are not expected to differ dramatically from instrument to instrument, but errors introduced at this level may have a strong effect on subsequent Raman data processing and interoperability, particularly for modelling purposes. Therefore, peak fitting analyses play a critical role in data comparison and reusability.^11–14^ Indeed, because of the large number of possible Raman instrument configurations in terms of type of spectrometer and optical path, it is generally accepted that data transfer requires preliminary harmonization.

In recent years, Raman peak fitting optimization has attracted close attention, associated with the strong development of Raman technology, particularly with the broad implementation of applications based on quantitative and qualitative multivariate data analysis, including the use of artificial intelligence and machine learning with massive amounts of data.^15^ Additionally, the extended use of handheld and portable Raman instruments, including small-footprint Raman analyzers^16–18^ with low resolution and signal-to-noise ratio (S/N), requires a closer look at the quality of the generated data and analytical procedures for spectral comparisons as well as model and library transfer. The use of the correct peak fitting shape during calibration dramatically improves reproducibility in these instruments.^14,19^ For Raman instruments with different spectrometers and/or optical collection paths, variations in parameters such as slit size or optical aberrations must be considered when analyzing the reference material spectra. Particularly for peaks at the edges of the charge-coupled device (CCD) detector these distortions can be high, and thus can cause deviations in peak analysis and, consequently, in the final calibration.

Calibration of the *x*-axis can be divided into two parts: position and resolution. The *x*-axis of a Raman spectrometer is usually calibrated using an emission spectrum (e.g., mercury, neon, or argon) and/or a Raman spectrum of a sample with at least one (e.g., silicon) or ideally several (e.g., toluene, polystyrene) well-known peaks. Calibration of the *x*-axis position with high accuracy requires the exact determination of the peak positions of the reference emission source spectrum to assign absolute wavelength/wavenumber values to the spectrometer pixels and obtaining the exact wavelength of the laser using the reference sample Raman spectrum to calculate the corresponding Raman shift–relative wavenumber values. The *x*-axis positions can also be determined with lower accuracy directly from the Raman spectrum. Regarding *x*-axis resolution calibration, it requires the exact determination of the peak widths, typically measured as the full width at half-maximum (FWHM, cm^–1^), from an emission spectrum and/or an adequate Raman spectrum such as that of calcite, with well-defined peaks, to assign resolution values to the pixels. The exact determination of both peak position and FWHM is done by peak fitting. This report systematically analyzes the effect of noise and peak fitting shape on the *x*-axis calibration steps in Raman spectroscopy along a broad range of instruments, focusing on well-established neon (Ne), silicon (Si), calcite (CaCO_3_), and polystyrene (PS) reference samples, and the implications on the harmonization of Raman spectra.

## Experimental

### Materials and Methods

**Figure 1.**
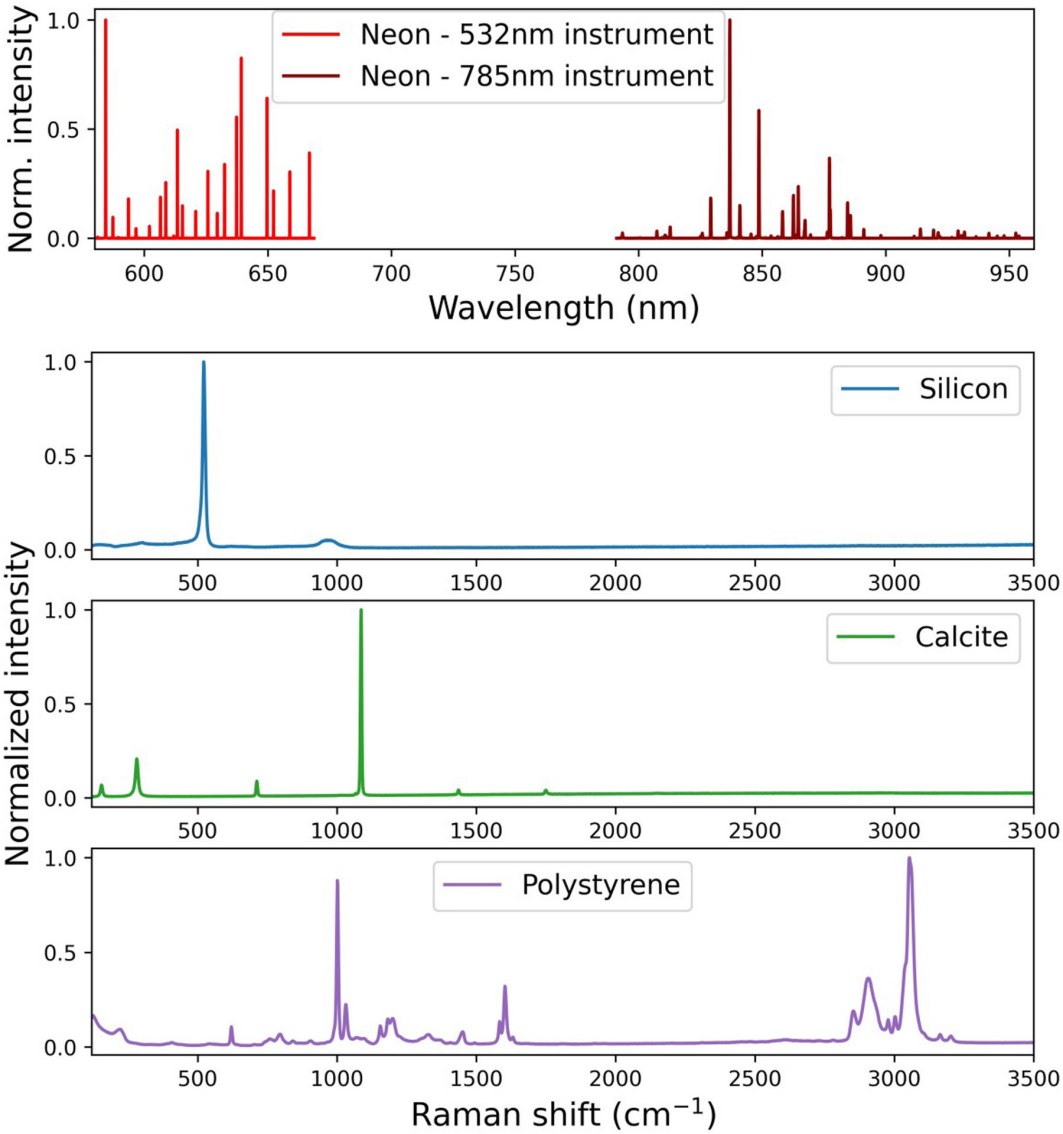
Reference material spectra. (Top) Ne emission lines recorded with a 532  nm (light red) and a 785  nm (dark red) laser configuration. (Below) Raman spectra of one of the Si samples (blue), calcite (green), and PS (purple).

This study uses four well-established reference samples for *x*-axis calibration in Raman spectroscopy: (i) Neon is suggested by many Raman manufacturers, with some Raman instruments having internal Ne lamps, as well as by ASTM E1840,^20^ E2529,^21^ and D7940^22^ for spectrometer verification and wavenumber calibration. Ne lamps are available from several suppliers, but often not certified. We used a Ne lamp supplied by ELODIZ (THEYA Ne calibration source) with the spectral line wavelengths traceable to NIST Atomic Spectra Database.^23^ (ii) Silicon is suggested by many manufacturers and in ISO 6775 for instrument readiness and Raman-shift quick verification.^24^ Moreover, Si samples are sometimes included within the instrument. However, Si is not available as a certified reference material (CRM). We analyzed five different Si materials supplied by Goodfellow Advanced Materials (UK). The Raman-shift-value obtained by Itoh and Shirono^25^ for NMIJ CRM 5606-a,^26^ a single-crystal, undoped Si material certified for positron defect measurements produced by the National Metrology Institute of Japan (NMIJ)^26^ was taken as a reference position for silicon. (iii) Polystyrene is suggested by ASTM E1840,^20^ among others, for wavenumber/Raman shift calibration, and by ASTM E1683^27^ for performance validation. PS CRM for Raman calibration was obtained from ELODIZ. (iv) Calcite is suggested by ASTM E2529^21^ for spectral resolution evaluation; calcite CRM was provided by ELODIZ. Example spectra of these samples are shown in Figure 1.

### Spectra Acquisition

Different sets of spectra were acquired with the 11 Raman instruments (S0 to S10) listed in Table S1 (Supplemental Material) to tackle the influence of noise and peak shapes.

To assess the influence of noise, 3458 spectra of boron-doped Si were recorded in S0^514^ with 1% laser power (0.12  mW) and 100  ms integration time.

To assess the influence of peak shape for fitting, ten Raman instruments (S1 to S10) from different manufacturers with laser wavelengths of 532, 633 and 785  nm and different optics were used to acquire spectra of four reference samples relevant for *x*-axis calibration. For each instrument, a Ne emission spectrum was acquired using an exposure time that resulted in an intensity of the highest peak of approximately 80% saturation. For some instruments using 532  nm excitation, a second Ne spectrum was acquired with a longer exposure time to enhance the small Ne emission peaks located in the range of 532 to 580  nm. Raman spectra of Si with different doping and crystal orientations, PS and calcite were also acquired. The laser power was set to moderate values to avoid sample burning (in the case of PS) or heating (in the case of Si), and the exposure time was set to obtain an intensity of the highest peak of approximately 80% saturation. The exact values were dependent on the instrument.

### Data Processing

The Si spectra acquired to tackle the influence of noise were cleaned from spikes with an Open Source algorithm^28^ developed in-house, based on peak prominence, and FWHM. No further pre-processing was required. Peak fitting of this spectra dataset was performed with the ramanchada2 Python library.^29^ For the peak shape study, the peaks of Ne emission spectra as well as of Si, calcite, and PS Raman spectra were fitted with different peak shapes using Altaxo^30^ with the following procedure:

- Spectra pre-processing: The SNIP algorithm^31^ with 40 iterations was used to subtract the baseline. The half-width was 15 cm^–1^ for Ne and 30 cm^–1^ for Si, calcite, and PS. The PS spectrum was cropped below a Raman shift of 100 cm^–1^. Raman spectra for portable and handheld devices (S1^532^, S2^532^, S3^532^, S8^785^, and S9^785^) are background subtracted.
- Peak finding to get the approximate positions, widths, and heights of the peaks: The peaks in the Ne, calcite and PS spectra were found with the continuous wavelet transform-based pattern matching method,^32^ whereas the peaks in the Si spectra were found by a simple topological search^33^ with a minimal prominence value of 1%.
- Peak fitting: The approximate peak positions, widths, and heights obtained in the peak-finding step were used to calculate the initial parameters for a nonlinear regression step (Levenberg–Marquardt) rendering refined values. Several shapes were used for the fitting model: Gaussian (normal distribution) and Lorentzian (Cauchy distribution) are the simplest, ideal, symmetrical peak shapes. Voigt and Pearson VII are also symmetric but have more parameters to be able to fit non-ideal peaks. Pearson IV is an asymmetric peak shape and, therefore, suitable to account for skewed peaks. Ne, Si, and calcite peaks were fitted individually using a range of the spectrum around the peak of twice the approximate FWHM. The many overlapping peaks in PS spectra were fitted together using a sum of up to 50 peaks. Since such large models tend to be unstable during nonlinear fitting, the Voigt and Pearson IV parameterizations were modified to improve the ability to constrain the fit parameters to reasonable values (see supplementary information).

To assess the fitting quality and select the most suitable peak shape for each sample, the fitting error of the relevant peak parameters (position and FWHM) was calculated as the standard deviation from the covariance matrix of the Levenberg–Marquardt fit and then averaged for each instrument and peak shape for comparison.

### Effect of Peak Fitting on the x-Axis Calibration

A Ne emission spectrum and a Si Raman spectrum were used to create multiple *x*-axis calibrations by combining the two best peak shapes for both. To evaluate the effect of these variations, the *x*-axis calibrations were then applied to a PS spectrum measured for each instrument. The procedure used to build and apply the *x*-calibration model is described in detail in the Supplemental Material. The performance of the calibration models was evaluated by comparing the positions of the four main peaks of the polystyrene spectrum with the ASTM E1840 values.

## Results and Discussion

### Influence of Noise

**Figure 2.**
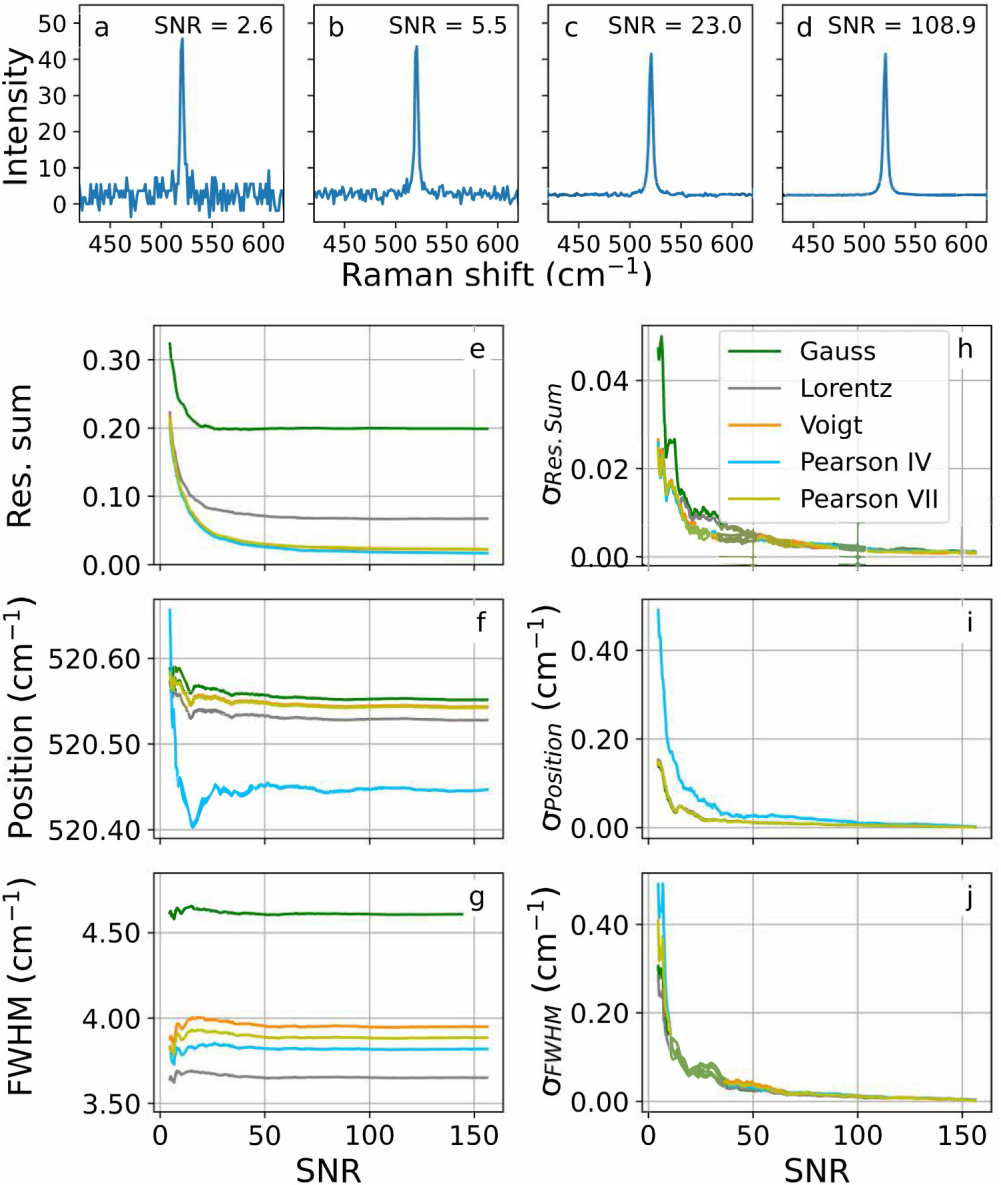
Silicon spectra with S/Ns of (a) 2.6, (b) 5.5, (c) 23.0 and (d) 108.9. Influence of the S/N on the (e) normalized fit residual sum (Res. Sum), (f) peak position, and (g) peak FWHM. (h–j) show the corresponding standard deviations.

To assess the influence of noise on the fitting parameters, from an initial dataset ( $S_{0}$ ) of 3458 boron-doped Si spectra acquired with the same integration time, a series of new datasets ( $S_{n}$ , where *n* = 1 to 15) was created using a cumulative averaging method, such that the *i*th spectrum in the new dataset, $S_{n,i}$ , is the average of all preceding spectra in the original dataset (Eq. 1):

(1) $$S_{n,i}=\;(1/i)\overset{i}{\underset{\;j=0}{\sum }}\;S_{0,j}$$

where i=1,2,…,3458 are the spectra indexes. This approach was repeated 15 times after random shuffling the original spectral dataset $S_{0}$ , meaning that the order in which the spectra are cumulatively averaged is different, to create different series $S_{n}$ to generate statistics. Therefore, the S/N, calculated as the height of the main peak of the spectrum divided by six times the standard deviation of the spectral intensity in an area of the spectrum with no Raman bands, increases with the spectrum index i in all the $S_{n}$ series. Examples of Si spectra with i = 0, 4, 104 and 1602, corresponding to S/N = 2.3, 5.3, 25 and 100, respectively, are plotted in that order in Figures 2a–d. The cumulative averaged spectra of the 15 new datasets were fit with Gaussian, Lorentzian, Voigt, Pearson IV and Pearson VII peak shapes making use of the *ramanchada2* Python library.^29^ Figure 2 also shows the (e) average of the fitting residual sum normalized to the peak height, (f) peak position, and (g) peak FWHM, as well as (h–j) their corresponding standard deviation.

Below an S/N of about 100, the standard deviations increase dramatically. Moreover, the fit peak positions show fluctuations below that value, which are especially intense for Pearson IV. This shape shows an offset with respect to the symmetric peak shapes. Consequently, peaks with an S/N below 100 should not be used to calibrate a Raman device. If the instrument would not allow better spectra, at least an S/N of 25 should be ensured for calibration; below that value the strong fluctuations in peak position and the high standard deviations may lead to inadequate calibration models.

### Influence of Peak Shapes

During calibration, peak fitting is used to determine peak position and FWHM in reference material spectra. Therefore, the resulting uncertainty and deviation from the true values of these parameters, which depend on the fitting function selected for each reference sample, are propagated throughout the following steps of the calibration. To assess this effect, five different peak shapes were chosen to fit Ne (used for *x*-axis position calibration and instrument resolution assessment), Si (used for *x*-axis position calibration), PS (used for Raman-shift *x*-axis calibration) and calcite (used for resolution assessment) peaks: Gaussian, Lorentzian, Voigt, Pearson VII, and Pearson IV.

**Figure 3.**
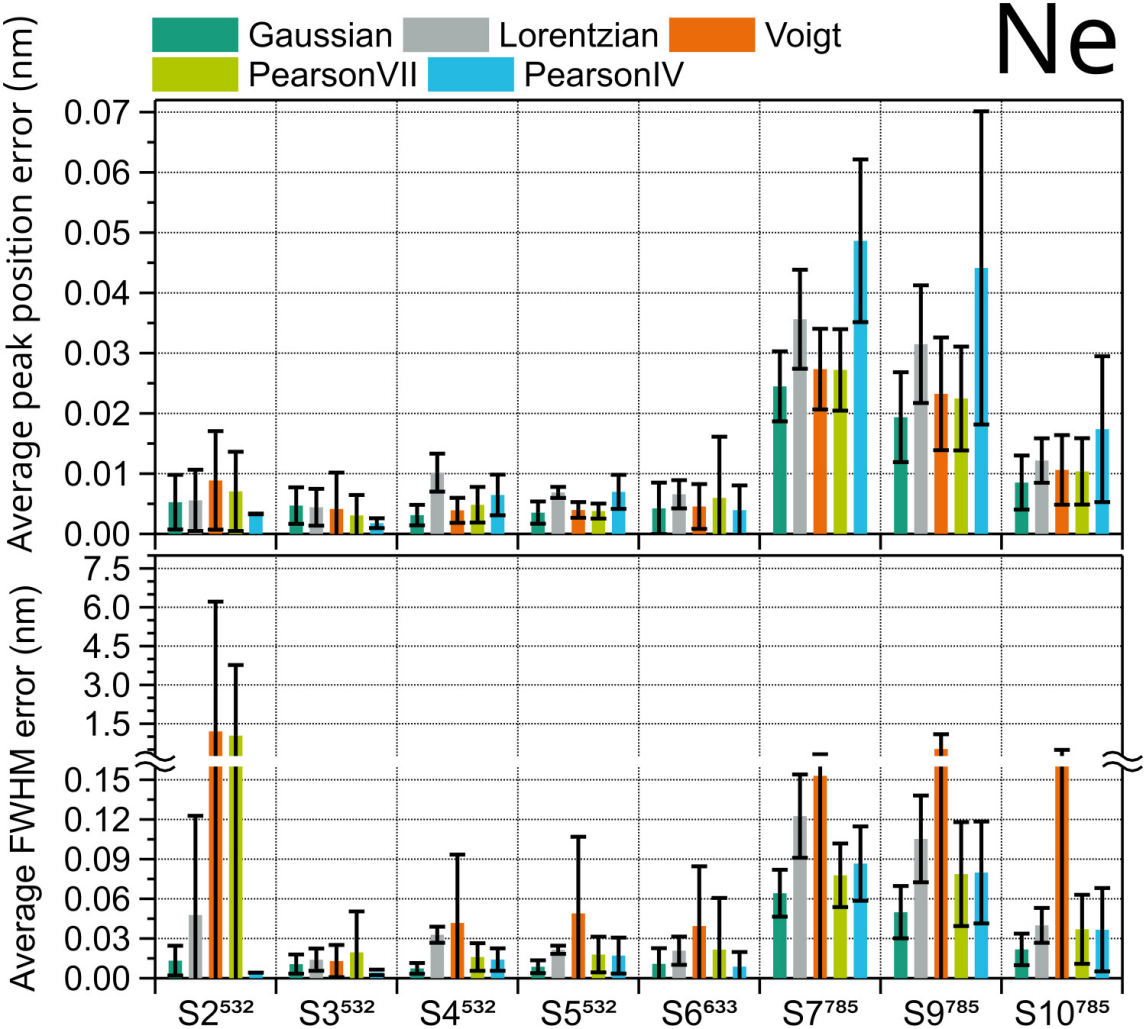
Average fit errors of peak position (top) and FWHM (bottom) of Ne emission spectrum peaks for eight different Raman instruments calculated using five peak shapes. Error bars are the standard deviation of approximately 25 Ne peaks.

To assess the appropriate fit shape for Ne spectra, the *x*-axis in Raman shift (cm^–1^) of eight Ne spectra taken with eight different instruments was converted to wavelength (nm) using the nominal laser excitation wavelength, which for the systems S1 to S4 is 532  nm, for S5 is 633  nm, and for S6 to S8 is 785  nm. Then, the Ne peaks were fitted using the five selected functions. For the Ne spectrum of each system, the fit errors (as evaluated from the covariance matrix of the nonlinear fit) of the peak position and FWHM of all matched peaks (approximately 25) were averaged for each peak shape and plotted in Figure 3. Five out of the eight systems show the lowest average fit error for both position and FWHM when using a Gaussian shape. Pearson IV is preferred in the remaining three systems (S2, S3, and S6), wherethe Gaussian shape has the second lowest fit error. Voigt function is the worst fitting option for Ne peaks, followed by the Lorentzian function. A ranking of the fitting shapes for Ne can be found in Table S2 of the Supplemental Material. Systems S7 and S9, both excited at 785  nm, presented the highest error, according to their significantly worse spectral resolution compared with the other Raman instruments.

**Figure 4.**
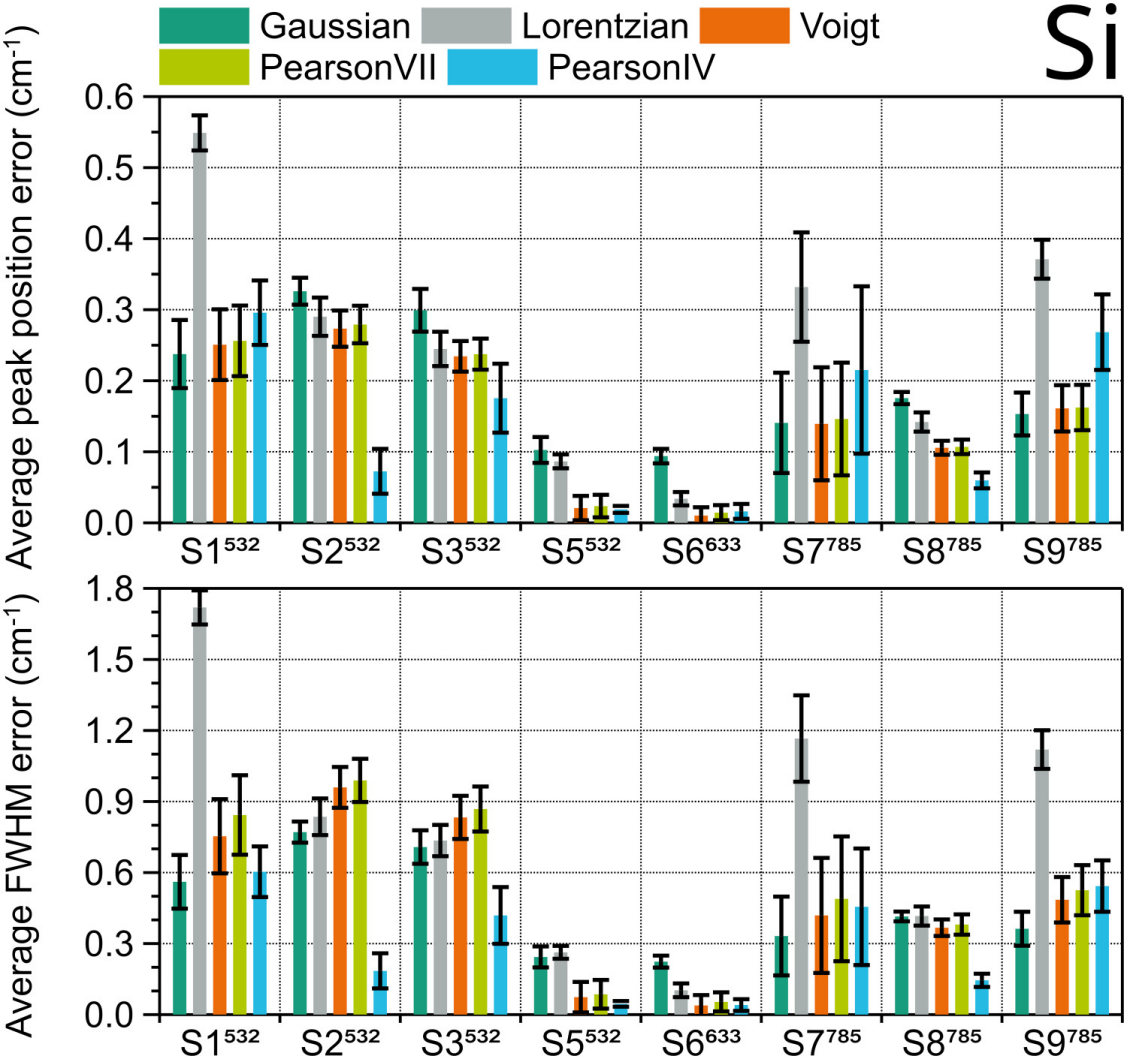
Average fit errors of position (top) and FWHM (bottom) of Si 520.14 cm^–1^ Raman peak in nine different systems, calculated using different peak shapes. Error bars are the standard deviation of 45 peaks (five samples × three measurement days × three measurements per day).

A similar procedure was used to evaluate the five fitting functions for the Si peak. As Si has just one peak, for each instrument, five Si samples with different dopant and crystal orientation were measured three times per day over three consecutive days without intermediate recalibration, to ensure maximum peak variability. The Si peak of the 45 spectra obtained with each instrument were fitted, and the fit errors of the peak position and FWHM were averaged for each peak shape and plotted in Figure 4. Lorentzian shape results in the highest average position and FWHM errors, so it must be avoided. Among the rest of the functions, the trend is not clear. Regarding peak position (Figure 4, upper diagram), which is the most relevant parameter for calibration, Voigt and Pearson IV seem to be the generally best fitting shapes. Pearson IV fit error is the lowest in the four systems (S8, S6, S5, and S3) with best spectral resolution (< 5 cm^–1^), and Voigt the second lowest, but Pearson IV fit error is also the second highest in the three systems with worst spectral resolution (> 8 cm^–1^), where Gaussian and Voigt have the best performance. This agrees with the results for the FWHM parameter (Figure 4, lower diagram). The corresponding ranking for Si can be found in Table S3 (Supplemental Material). Therefore, for any type of silicon sample used in instruments with unknown spectral resolution Voigt fitting is a safe choice, but for highest accuracy in systems with high spectral resolution Pearson IV would be the optimal fitting shape.

**Figure 5.**
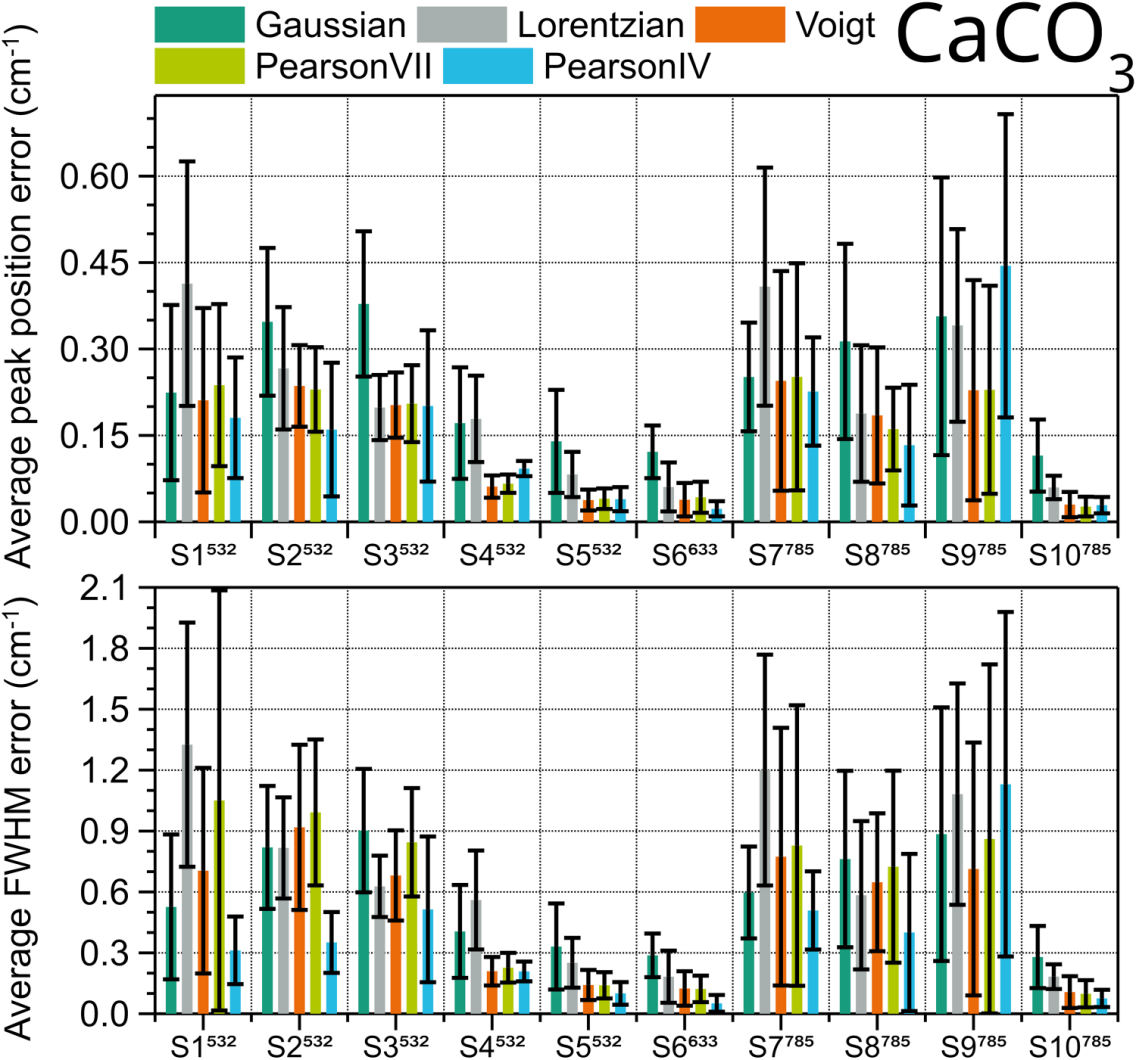
Average fit errors of position (top) and FWHM (bottom) of calcite peaks at 155.5, 282, 711.8, 1085, and 1435.6 cm^–1^ obtained in ten different systems, calculated using different peak shapes. Error bars are the standard deviation of the five peaks.

**Figure 6.**
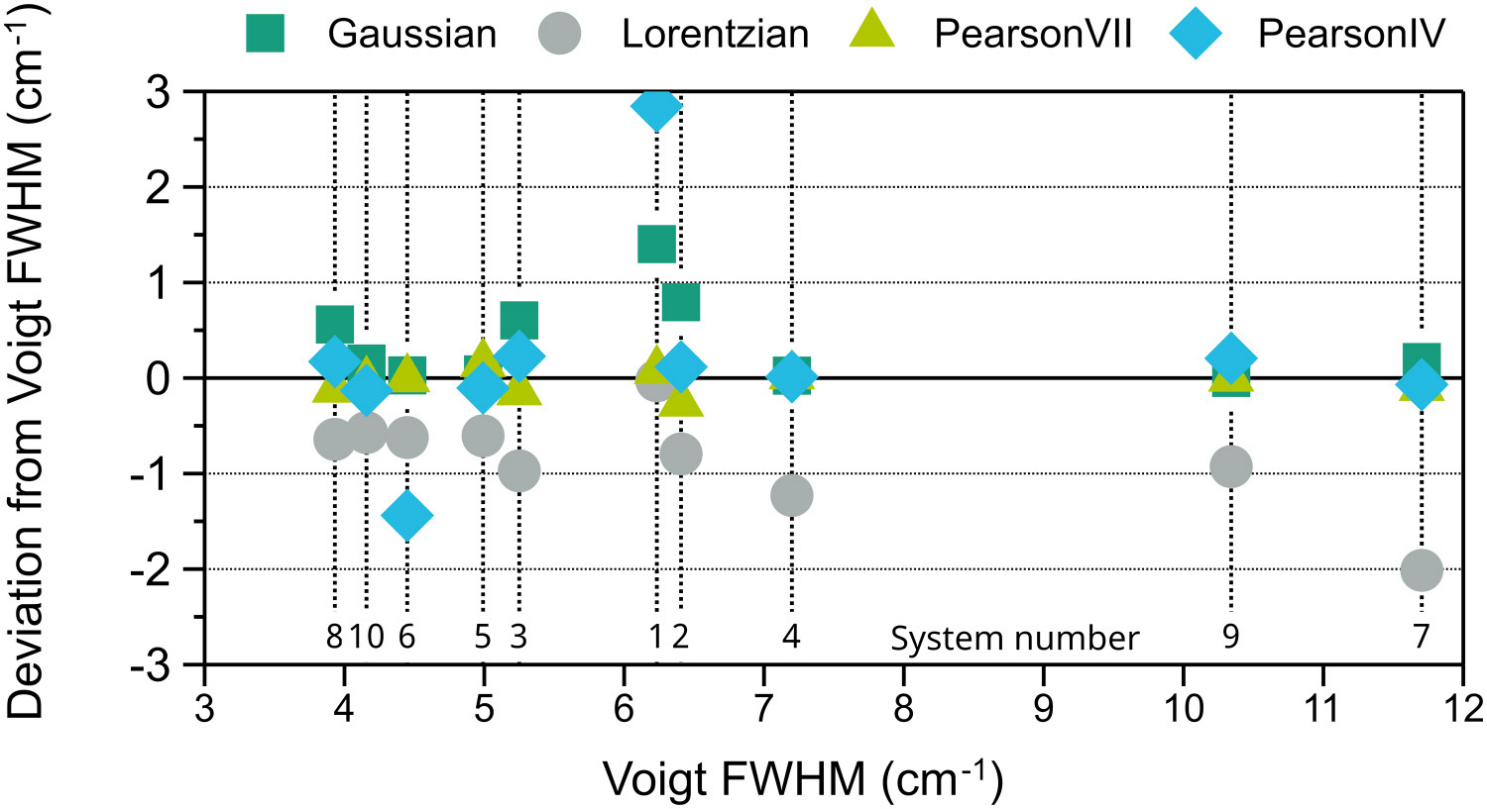
Deviation of the FWHM values obtained with Gaussian, Lorentzian, Pearson VII, and Pearson IV fitting from the value obtained with Voigt fitting for the 1085 cm^–1^ calcite peak versus the Voigt FWHM value. The numbers at the bottom of the graph designate the system.

The same procedure was repeated with the five peaks from the spectra of calcite. The results of the evaluation are shown in Figure 5. For both peak position and FWHM Pearson IV is the best fitting type, with the lowest error in eight out of ten systems. The two systems for which Voigt is better than Pearson IV, one using 532  nm excitation (S4) and the other one 785  nm (S9), have relatively low spectral resolution, i.e., high FWHM (7.2 cm^–1^ and 10.3 cm^–1^, see Figure 6). On the contrary, in S5, S6, and S10, the three instruments with better pixel resolution (≤ 1.3 cm^–1^) and three of the four instruments with better spectral resolution, each one with a different excitation wavelength, both Voigt and Pearson IV functions perform similarly well, and the errors are the lowest among all the instruments. Therefore, spectra taken with worse pixel resolution, related to a lower number of points defining the peak, tend to be better fitted with Pearson IV, with the exception of S4. However, the Voigt function is suggested to fitthe calcite peak at 1085 cm^–1^ in ASTM E2529 to evaluate with the FWHM value the resolution of instruments that use 785  nm excitation. In the spectra collected with the instruments considered in this interlaboratory study, the FWHM of this peak fitted with Voigt ranges between 3.9 and 11.7 cm^–1^. The FWHM value deviation of other fitting shapes with respect to the Voigt fitting FWHM value for the 1085 cm^–1^ peak is shown in Figure 6.

The Lorentzian and Gaussian fittings, which are consistent with the shape of Cauchy and normal distributions, respectively, consistently provide lower (the former) and higher (the latter) FWHM values, so their use for calcite should be discarded. The value deviation of the other fittings is lower than ± 0.2 cm^–1^. Pearson VII has both positive and negative deviations never exceeding a magnitude of 0.26, but is never the best option. Generally, Pearson IV has also a very low deviation from the Voigt FWHM, except for two systems, one with negative and the other one with positive large deviations.

Overall, this indicates that the Pearson IV peak fitting shape is preferable for calcite peak FWHM. However, as its behavior compared to the Voigt peak fitting shape is erratic, it is not possible to adjust the equation from ASTM E2529 to be used with Pearson IV. The corresponding ranking for calcite can be found in Table S4 (Supplemental Material).

**Figure 7.**
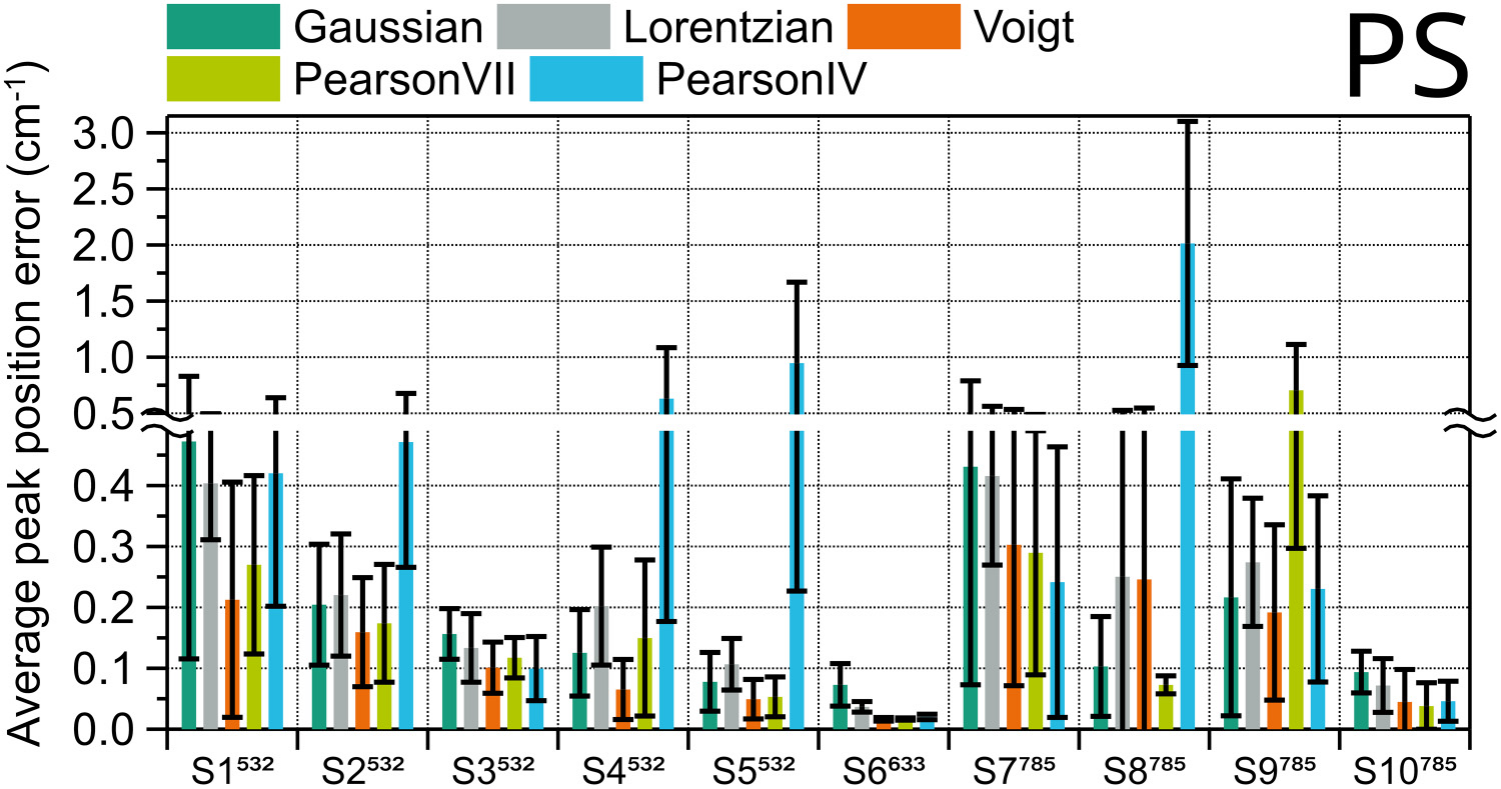
Average position fit error of four polystyrene peaks in ten different systems, calculated using different peak shapes. Error bars are the standard deviation.

Polystyrene is one of the materials listed in ASTM E1840^20^ and the main pharmacopeias^1^ to calibrate the Raman shift *x*-axis. As such, the peak position assessment must be as precise as possible. However, no guidance is provided in these documents on how to perform the peak fitting. The certificate of the PS CRM provided by the Japanese metrology institute (NMIJ RM 8158-a)^34^ indicates that the mixed Gaussian–Lorentzian (pseudo-Voigt) function was used to fit PS peaks, including 1448.4, 2906.2, and 3055.1 cm^–1^, which are bilaterally asymmetric, whereas no information can be extracted from the certificate of the PS CRM provided by the Chinese metrology institute (NIM GBW13664).^35^ Here, four prominent peaks commonly referred to in these guides (620.9 cm^–1^, 1001.4 cm^–1^, 1602.3 cm^–1^, 3054.3 cm^–1^) were fitted with the five selected functions, and then the average values of the position and FWHM errors were calculated, as with previous samples. The results are presented in Figure 7 and Figure S3 (Supplemental Material), with the error bars representing the standard deviation of the four peaks.

Voigt is the best fitting function for the position in most instruments, six out of ten; Pearson IV and VII are better each in two of the other instruments. In the instances where Pearson IV and VII are preferred, though, Voigt’s error is very similar (S3, S7, S10), except for the instrument with highest spectral resolution, S8^785^, so Voigt should be the fitting of choice. The FWHM results in Figure S1 (Supplemental Material) have a very similar trend. The corresponding ranking for PS can be found in Table S6 (Supplemental Material).

**Figure 8.**
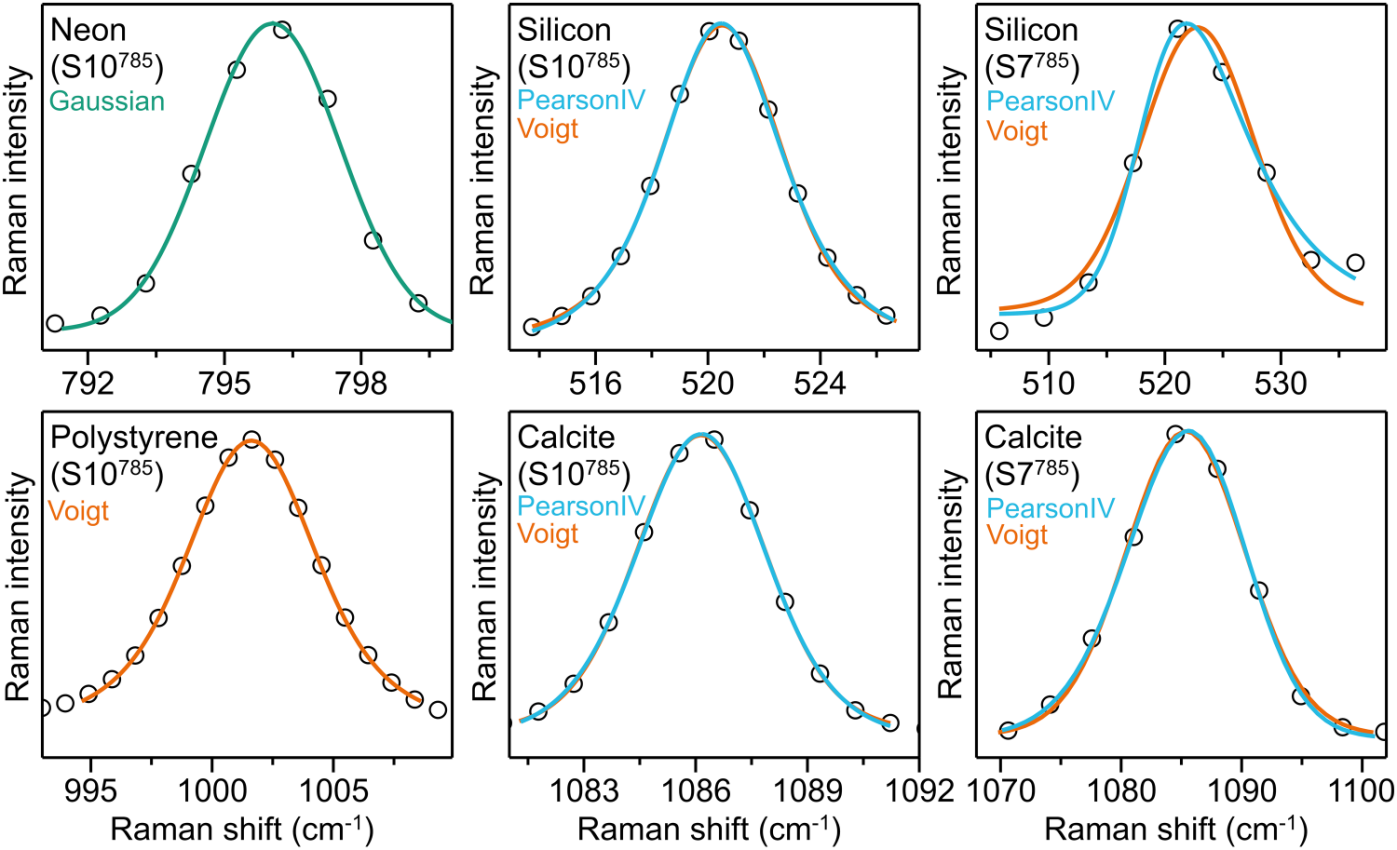
Examples of relevant Raman peaks of four reference material spectra. Black circles show the experimental data, colored lines show the fit curves for each material: On the left column, Ne and PS peaks are best fitted with Gaussian (green) and Voigt (orange), respectively. For silicon and calcite, spectra obtained in high spectral resolution systems like S10^785^ (middle column) are well fitted with both Voigt and Pearson IV (cyan), whereas with low resolution systems like S7^785^ (right column) Pearson IV tends to provide a better fitting, particularly for the silicon peak. Both fittings are shown for comparison.

Example spectra of the different reference materials with their main peak fitted with the corresponding optimal shape as derived from this study are shown in Figure 8. Additionally, for silicon and calcite both Voigt and Pearson IV fit curves are plotted for a direct comparison.

Based on these results, we have assessed the influence of the peak shapes used for Ne and Si on the quality of the *x*-calibration model. For each of the ten systems, four different calibration models have been built, using Gaussian and Pearson IV fitting shapes for the Ne spectra and Pearson IV and Voigt for the Si spectra. The four *x*-calibration models were applied to a spectrum of PS, and the resulting four *x*-calibrated PS spectra were then fitted with the Voigt shape function. The quality of the four calibration models was assessed by evaluating the position deviations of the four PS peaks from their respective ASTM positions. A score was given for each peak based on the σ values of ASTM E1840, scoring 3 if it is within 1σ, 2 if within 2σ, and 1 if within 3σ. If any peak was above 3σ from the ASTM value, the calibration was marked as a failure. In Table I, the sum of the scores of the four PS peaks are shown for the different Raman systems. Overall, Gaussian for Ne and Pearson IV for Si lead to the best results, with all systems having the four PS peaks checked within the ASTM 3σ boundaries. However, only two systems got a perfect score of 12, with all peaks within 1σ. Changing Si fitting for Voigt provided a higher score for three systems and a lower score just in one but failed for system 7. Combinations where Pearson IV was used for Ne should be discarded, as they failed not only for system 7 but also for system 9; moreover, no system achieved a perfect score.

**Table I.** Scores of the four Ne + Si calibrations obtained with different peak fitting shape combinations. The *x*-calibrations were applied to PS spectra obtained with different instruments and four prominent PS peaks were fitted with Voigt shape to calculate their calibrated positions, the differences with respect to the corresponding ASTM E1840 positions, and if they fall within 1σ (3 points), 2σ (2 points), 3σ (1 point), or beyond (fail). The total score value provided is the sum of the scores of the four PS peaks.

Ne	Gaussian		Pearson IV	
Si	Pearson IV	Voigt	Pearson IV	Voigt
S1^532^	12	10	10	10
S2^532^	9	12	11	10
S3^532^	12	12	11	10
S4^532^	10	12	9	10
S6^633^	9	9	9	9
S7^785^	7	Fail	Fail	Fail
S8^785^	12	12	10	11
S9^785^	8	8	Fail	Fail
S10^785^	11	11	9	9
Sum	90	86	69	69
Passed	100%	89%	78%	78%
<1σ	33%	44%	0%	0%

## Conclusion

Raman peaks used for wavenumber calibration of a Raman instrument should have a S/N of at least 100 to ensure accurate position calculation by fitting. The optimal distribution function for peak fitting in reference material spectra for calibration depends on the analyzed reference material. Since the natural line shape in optical emission spectra from sources with extremely narrow lines, such as Ne, cannot be resolved, the peak shape is predominantly determined by the spectrometer optics, resulting in a Gaussian profile. The Raman spectra must be analyzed on a case-by-case basis. For *x*-calibration using a Si sample, as the peak position is required and the peak shape is often slightly asymmetric, Pearson IV should be used for high spectral resolution instruments, because symmetric peak shapes may show an offset, but Voigt might be better for low or unknown spectral resolution instruments. Calcite should be fitted with a Voigt line shape, as ASTM E2529 recommends, because the error in peak position and peak width is similar to that obtained with Pearson IV and the results are less erratic, however, some instruments with bad pixel resolution may require to be fitted with Pearson IV. PS peak positions, used for *x*-axis Raman shift calibration, show the lowest error when using Voigt fitting.

## Supplemental Material

sj-docx-1-asp-10.1177_00037028251330654 - Supplemental material for An Interlaboratory Study to Minimize Wavelength Calibration Uncertainty Due to Peak Fitting of Reference Material Spectra in Raman Spectroscopy Supplemental material, sj-docx-1-asp-10.1177_00037028251330654 for An Interlaboratory Study to Minimize Wavelength Calibration Uncertainty Due to Peak Fitting of Reference Material Spectra in Raman Spectroscopy by Dirk Lellinger, James Thomson, Nicolas Coca-Lopez, Afroditi Ntziouni, Nikolaos Nikoloudakis, María Fernández-Álvarez, Nina Jeliazkova, Miguel A. Bañares, Raquel Portela and Enrique Lozano Diz in Applied Spectroscopy
