# Supplementary material for: Interlaboratory Study to Minimize Wavelength Calibration Uncertainty Due to Peak Fitting of Reference Material Spectra in Raman Spectroscopy
Source: Appl Spectrosc. 2025 Apr 24;79(12):1669–79. doi: 10.1177/00037028251330654 (PMC12634889; doi:10.1177/00037028251330654)
Supplement: sj-docx-1-asp-10.1177_00037028251330654 - Supplemental material for An Interlaboratory Study to Minimize Wavelength Calibration Uncertainty Due to Peak Fitting of Reference Material Spectra in Raman Spectroscopy [file sj-docx-1-asp-10.1177_00037028251330654.docx]

**Supplemental Material**

**An Interlaboratory Study to Minimize Wavelength Calibration Uncertainty Due to Peak Fitting of Reference Material Spectra in Raman Spectroscopy**

Dirk Lellinger^1^*, James Thomson^2^, Nicolas Coca-Lopez^3^, Afroditi Ntziouni^4^, Nikolaos Nikoloudakis^4^, María Fernández-Álvarez^5^, Nina Jeliazkova^6^, Miguel A. Bañares^3^, Raquel Portela^3^*, Enrique Lozano Diz^2^*

^1^Fraunhofer LBF, Darmstadt, Germany

^2^ ELODIZ Ltd., High Wycombe, UK

^3^ Instituto de Catalisis y Petroleoquimica (ICP), CSIC, Madrid, Spain

^4^ NTUA, Athens, Greece

^5^ Instituto de Ceramica y Vidrio (ICV), CSIC, Madrid, Spain, ^6^ Ideaconsult Ltd., Bulgaria,

* Corresponding author emails: dirk.lellinger@lbf.fraunhofer.de, raquel.portela@csic.es, enriquel@elodiz.com

ContentS

**Description of the Raman systems 2**

**Procedure to build and apply an x-calibration model 2**

Building the x-axis wavelength calibration with Ne 2

Ne peak matching 2

Calculation of the spectrometer pixel wavelength 3

Estimating the laser wavelength with Si to calculate Raman shift 4

Applying the x-calibration 5

**Reparameterization of Voigt and Pearson IV functions 5**

**Peak fitting shapes ranking 6**

**Average error of the fit FWHM of polystyrene 7**

**Description of the Raman Systems**

**Table S1.** Description of the Raman instruments used to obtain the spectra. The superscript in the shortcut is the nominal excitation laser wavelength in nm. The spectral resolution is obtained from the FWHM of the 1085 cm^-1^ calcite peak fitted with Voigt shape using the ASTM2529 formula: Spectral Resolution$=(FWHM-0.684 \mathrm{cm}^{-1})/1.0209$. The pixel resolution is obtained from the mean difference of the shift values of two adjacent pixels of the entire spectrum.

| **Shortcut** | **Manufacturer** | **Model** | **Spectral resolution** | **Pixel resolution** | **Measurement configuration** |
| --- | --- | --- | --- | --- | --- |
| S0^514^ | Renishaw | inVia Qontor | 4.8 cm^-1^ | 1.4 cm^-1^ | Single-shot  Center at 510.31 cm^-1^ |
| S1^532^ | Elodiz | Neegala | 5.44 cm^-1^ | 2.86 cm^-1^ | Single-shot  Center at 3149.06 cm^-1^ |
| S2^532^ | BWTEK | iRaman | 5.60 cm^-1^ | 2.15 cm^-1^ | Single-shot  Center at 2442.76 cm^-1^ |
| S3^532^ | BWTEK | iRaman | 4.47 cm^-1^ | 2.11 cm^-1^ | Single-shot  Center at 2534.80 cm^- 1^ |
| S4^532^ | WITec | Alpha 500 | 6.38 cm^-1^ | 2.38 cm^-1^ | Single-shot  Centered at 2150 cm^-1^ |
| S5^532^ | Renishaw | inVia Reflex | 4.22 cm^-1^ | 1.32 cm^-1^ | Moving grating |
| S6^633^ | Horiba | LabRam | 3.69 cm^-1^ | 0.73 cm^-1^ | Moving grating |
| S7^785^ | Horiba | LabRam | 10.80 cm^-1^ | 3.06 cm^-1^ | Moving grating |
| S8^785^ | BWTEK | iRaman | 3.18 cm^-1^ | 1.72 cm^-1^ | Single-shot  Center at 1856.96 cm^-1^ |
| S9^785^ | Zolix | FinderEdge | 9.46 cm^-1^ | 1.67 cm^-1^ | Single-shot  Center at 2086 cm^-1^ |
| S10^785^ | Renishaw | inVia Reflex | 3.40 cm^-1^ | 0.79 cm^-1^ | Moving grating |

# Procedure to build and apply an x-calibration model

## Building the *x*-Axis Wavelength Calibration with Ne

### Ne Peak Matching

The peak positions found in the measured neon spectrum are matched with the corresponding positions obtained from the NIST Atomic Spectra database^17^ in the following steps:

- Convert the found non-calibrated peak positions in Raman shift (*S_nc_*, in cm^-1^) to non-calibrated wavelength positions ($\lambda_{\mathrm{nc}},$nm) using the nominal (thus approximate) wavelength of the laser provided by the manufacturer ($\lambda_{Laser,approx}$) in Eq. S1 for Ne.

$\lambda_{\mathrm{nc}}=\frac{1}{{\lambda_{\mathrm{Laser},\mathrm{approx}}}^{-1}-{10}^{-7} s_{nc}}$ Eq. S1

- Pair each of the Ne found peak positions with each of the positions in the NIST database that are in a range of about 30 nm wavelength around the found position. This results in a list of value pairs, one in the spectrometer (uncalibrated) domain and one in the NIST (calibrated) domain.

- Choose arbitrarily two pairs from the list to obtain an initial linear dependency between the NIST calibrated and the spectrometer uncalibrated wavelength domains.

- For each peak in NIST database, calculate the linearly correlated position in the spectrometer domain and get the corresponding intensity at this point in the measured Ne spectrum.

- Sum up the intensities for all NIST peaks.

- Repeat the procedure for all possible two-pair combinations within the list of pairs.

- The combination for which the sum of intensities is the highest is used as a provisional linear dependency to obtain for each NIST peak (calibrated wavelength domain) the corresponding uncalibrated wavelength in the spectrometer domain.

- If a measured peak was found in the vicinity (within one FWHM of the found peak) of the NIST peak position in the uncalibrated spectrometer wavelength domain they form a match. If more than one peak was found in the vicinity, this peak is not used for matching.

### Calculation of the Spectrometer Pixel Wavelength

The list of Ne peak matches is used to calculate the calibrated wavelength of the spectrometer pixels by interpolation, using smoothing polyharmonic spline functions with a derivative order of 2 and a regularization parameter of 50. The procedure is the following:

- calculate a first interpolation curve, INTERPOLATION1: *x*-values = linearly correlated NIST position, *y*-values = difference between measured position and NIST position. This is visualized in Figure S1.


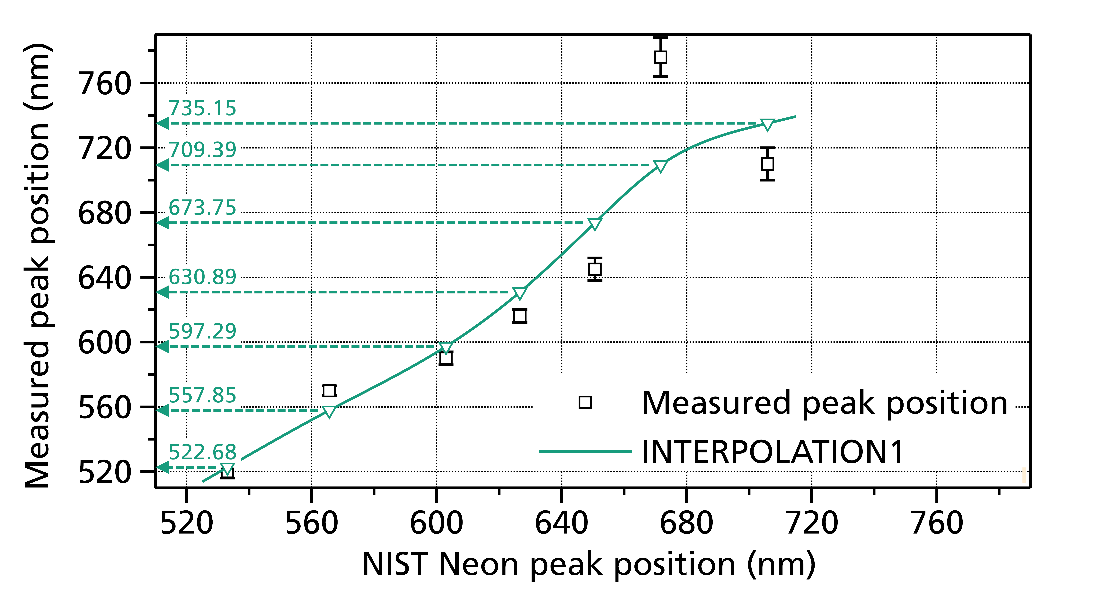


**Figure S1.** Construction of INTERPOLATION1 by plotting the neon measured peak positions versus the corresponding NIST peak positions that are a match according to 2.1.1. (black squares; the error bars designate the error of the fit parameter) and then interpolating the values with a smoothing polyharmonic spline function (green line). For the sake of clarity, in this figure: (i) the deviations and error bars were greatly exaggerated, (ii) only 7 peak positions are shown (out of approx. 25), and (iii) the measured peak positions were interpolated instead of the deviations between measured and NIST peak positions, which is the parameter effectively interpolated, as described above. Note: The y-values of the interpolation at the NIST positions (green labels at the left side) are then used to create INTERPOLATION2 according to Figure S2.

- Use INTERPOLATION1 to transform the NIST position of each match into the position in the spectrometer domain, and use these positions as x-values for calculation of INTERPOLATION2. The y-values of INTERPOLATION2 are the difference between interpolated NIST position and measured position, see Figure S2.


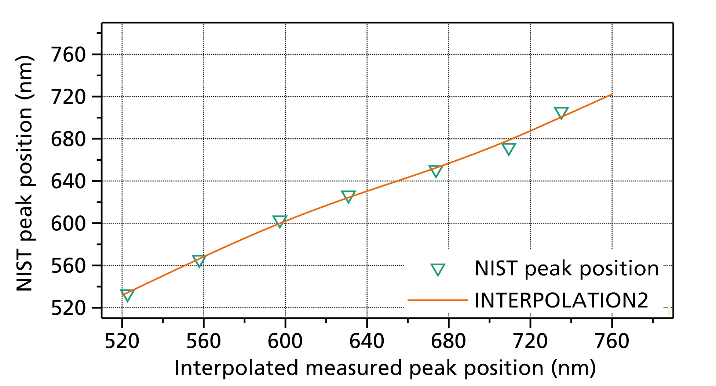


**Figure S2.** Construction of INTERPOLATION2 by plotting the NIST peak positions versus the interpolated measured positions from INTERPOLATION1 (green labels at the left side in Figure S1) and then interpolating them with a smoothing polyharmonic spline function. Note: INTERPOLATION2 is used to transform the wavelength of each pixel of the spectrometer to the calibrated wavelength using Eq. S2 with the approximate laser wavelength.

- INTERPOLATION2 is used to transform the wavelength of each pixel of the spectrometer to the calibrated wavelength with Eq. S2 for Ne:

$\lambda_{\mathrm{cal}}=\lambda_{\mathrm{nc}}+\mathrm{INTERPOLATION}2(\lambda_{\mathrm{nc}})$ Eq. S2

## Estimating the Laser Wavelength with Si to Calculate Raman Shift

The difference between the found position of the silicon peak and the reference position published in the literature, $s_{Si,ref}=520.25 \mathrm{cm}^{-1}$,^19^ is used to estimate the real laser wavelength, $\lambda_{Laser,est}$. First, the found peak position of Si (non-calibrated shift) is converted to non-calibrated wavelength using the Eq. S1 for Si. Secondly, the non-calibrated wavelength is calibrated using INTERPOLATION2 and Eq. S2 for Si. Finally, $\lambda_{Laser,est}$ is calculated with Eq. S3.$\lambda_{Laser,est}$ is then used to transform calibrated wavelength into calibrated Raman shift values with Eq. S4:

$\lambda_{\mathrm{Laser},est}=\frac{1}{{\lambda_{\mathrm{Si},\mathrm{cal}}}^{-1}-{10}^{-7} s_{\mathrm{Si},\mathrm{ref}}}$ Eq. S3

$s_{cal}={10}^{7}\left[ \frac{1}{\lambda_{\mathrm{Laser},est}}-\frac{1}{\lambda_{\mathrm{cal}}} \right]$ Eq. S4

## Applying the *x*-Calibration

Each uncalibrated shift value $s_{nc}$ (cm^-1^) of any measured spectra (in this work PS and calcite spectra) is calibrated by using first Eq. S1 with $\lambda_{Laser,approx}$ to obtain the uncalibrated wavelength value $\lambda_{\mathrm{nc}}$(nm), then Eq. S2 with INTERPOLATION2 to obtain the calibrated wavelength value $\lambda_{c}$(nm), and finally Eq. S4 with $\lambda_{Laser,est}$ to obtain the calibrated shift value $s_{cal}$ (cm^-1^).

# Reparameterization of Voigt and Pearson IV functions

The typical parameterization for the Voigt function is $\mathrm{Voigt}\left( x,{A, x}_{c},\sigma, \gamma\right)$, in which $A$ is the area under the peak, $x_{c}$ is the location of the peak maximum, $\sigma$ is the broadness parameter of the underlying Gaussian distribution and $\gamma$ is the broadness parameter of the underlying Lorentzian distribution. However, this parametrization is not well suited for fits in which the FWHM of the peak has to be constrained, because both $\sigma$ and $\gamma$ influence the FWHM of the function, making a constraint on the FWHM impossible. Thus, for this paper, another parametrization was used, $\mathrm{Voigt}\left( x,{A, x}_{c},w,\upsilon\right)$, for which the relation to the original parameters is:

$\sigma=w \sqrt{\frac{\upsilon}{\ln4}}$ Eq. S5

$\gamma=w (1-\upsilon)$ Eq. S6

With this reparameterization, the FWHM of the Voigt function is approximately $2w$ (with an error of max. 3%). This accuracy is sufficient to effectively constrain the FWHM parameter of the function. The range of the parameter $\upsilon$ is [0, 1], with the limits $\upsilon=0$ for a Lorentzian and $\upsilon=1$ for a Gaussian shape. Implementation details can be found in the source code of Altaxo:

https://github.com/Altaxo/Altaxo/blob/master/Altaxo/Core/Calc/FitFunctions/Probability/VoigtAreaParametrizationNu.cs

The same applies to the Pearson IV function, which is originally defined as:

$\mathrm{PearsonI}V_{\mathrm{Original}}\left( x,{a, x}_{0},\sigma,m,n \right)={a \left( 1+z^{2} \right)}^{-m}\exp\left( -n\arctan\left( z \right) \right)$ Eq. S7

$z=\frac{x-x_{0}}{\sigma}$ Eq. S8

This original parameterization poses three issues: (i) the height parameter $a$ does not represent the maximum peak value, (ii) the location parameter $x_{0}$ does not represent the location of the maximum of the peak, and (iii) the FWHM of the peak cannot be easily related to the broadness parameter $\sigma$ , but also depends on parameters $m$ and $n$.

The reparameterization was considerably more complicated compared to the Voigt function, because 4 new parameters were chosen: $h$ represents the height of the peak maximum, $x_{c}$ is the location of the peak maximum, $w$ is approximately the FWHM of the peak, and $\nu$ determines the skewness. The parameter $m$ is still the exponent of the Pearson IV function.

$\mathrm{PearsonIV}\left( x,h,x_{c},w,m,\nu\right)=h\left( \frac{1+z^{2}}{1+\nu^{2}} \right)^{-m}\exp\left( -2m\nu\left[ \arctan\left( z \right)+\arctan\left( \nu\right) \right] \right)$ Eq. S9

$z=\sqrt{\left( 2^{1/m}-1 \right)\left( 1+\nu^{2} \right)}\left( \frac{x-x_{c}}{w} \right)-v$ Eq. S10

The error for the constraint of the FWHM is about 38%, but again is sufficient to be used for effectively constraining the FWHM of the peak. Implementation details can be found in the source code of Altaxo:

https://github.com/Altaxo/Altaxo/blob/master/Altaxo/Core/Calc/FitFunctions/Peaks/PearsonIVAmplitudeParametrizationHPW.cs

# Peak fitting shapes ranking

To build a ranking, scores were assigned to the fits on a per system basis. It was assumed that the peak shape providing the lowest average fit error of the parameter (either position or FWHM) for that system is the best fit, thus it receives a score of 5. In contrast, the highest average error for that system results in the worst fit, and thus receives a score of 1. The scores of all systems were summed-up in order to assess the best fit shape across all systems.

**Table S2.** Peak fitting shapes ranking for Ne peaks position and FWHM according to the average errors per system.

| **Ne** | S2^532^ | | S3^532^ | | S4^532^ | | S5^532^ | | S6^633^ | | S7^785^ | | S9^785^ | | S10^785^ | | Score sum | |
| --- | --- | --- | --- | --- | --- | --- | --- | --- | --- | --- | --- | --- | --- | --- | --- | --- | --- | --- |
|  | position | FWHM | position | FWHM | position | FWHM | position | FWHM | position | FWHM | position | FWHM | position | FWHM | position | FWHM | position | FWHM |
| Gaussian | 4 | 4 | 1 | 4 | 5 | 5 | 5 | 5 | 4 | 4 | 5 | 5 | 5 | 5 | 5 | 5 | 34 | 37 |
| Lorentzian | 3 | 3 | 2 | 2 | 1 | 2 | 2 | 2 | 1 | 3 | 2 | 2 | 2 | 2 | 2 | 2 | 15 | 18 |
| Voigt | 1 | 1 | 3 | 3 | 4 | 1 | 3 | 1 | 3 | 1 | 3 | 1 | 3 | 1 | 3 | 1 | 23 | 10 |
| Pearson IV | 5 | 5 | 5 | 5 | 2 | 4 | 1 | 4 | 5 | 5 | 1 | 3 | 1 | 3 | 1 | 4 | 21 | 33 |
| Pearson VII | 2 | 2 | 4 | 1 | 3 | 3 | 4 | 3 | 2 | 2 | 4 | 4 | 4 | 4 | 4 | 3 | 27 | 22 |

**Table S3a.** Peak fitting shapes ranking for Si peaks position according to the average errors per system.

| **Si** (position) | S1^532^ | S2^532^ | S3^532^ | S5^532^ | S6^633^ | S7^785^ | S8^785^ | S9^785^ | Score Sum |
| --- | --- | --- | --- | --- | --- | --- | --- | --- | --- |
| Gaussian | 5 | 1 | 1 | 1 | 1 | 4 | 1 | 5 | 19 |
| Lorentzian | 1 | 2 | 2 | 2 | 2 | 1 | 2 | 1 | 13 |
| Voigt | 4 | 4 | 4 | 4 | 5 | 5 | 4 | 4 | 34 |
| Pearson IV | 2 | 5 | 5 | 5 | 3 | 2 | 5 | 2 | 29 |
| Pearson VII | 3 | 3 | 3 | 3 | 4 | 3 | 3 | 3 | 25 |

**Table S3b.** Peak fitting shape weighed ranking for Si peaks position according to the average errors per system.

| **Si** (position, weighed) | S1^532^ | S2^532^ | S3^532^ | S5^532^ | S6^633^ | S7^785^ | S8^785^ | S9^785^ | Score Sum |
| --- | --- | --- | --- | --- | --- | --- | --- | --- | --- |
| Gaussian | 5.0 | 1.0 | 1.0 | 1.0 | 1.0 | 5.0 | 1.0 | 5.0 | 20.0 |
| Lorentzian | 1.0 | 1.6 | 2.8 | 1.9 | 3.9 | 1.0 | 2.2 | 1.0 | 15.3 |
| Voigt | 4.8 | 1.8 | 3.1 | 4.8 | 5.0 | 5.0 | 3.4 | 4.9 | 32.8 |
| Pearson IV | 4.3 | 5.0 | 5.0 | 5.0 | 4.7 | 3.4 | 5.0 | 2.9 | 35.3 |
| Pearson VII | 4.8 | 1.7 | 3.0 | 4.7 | 4.8 | 4.9 | 3.4 | 4.8 | 32.1 |

**Table S4.** Peak fitting shape ranking for calcite peaks position according to the average errors per system.

| **Calcite** (position) | S1^532^ | S2^532^ | S3^532^ | S4^532^ | S5^532^ | S6^633^ | S7^785^ | S8^785^ | S9^785^ | S10^785^ | Score Sum |
| --- | --- | --- | --- | --- | --- | --- | --- | --- | --- | --- | --- |
| Gaussian | 3 | 1 | 1 | 2 | 1 | 1 | 2 | 1 | 2 | 1 | 15 |
| Lorentzian | 1 | 2 | 5 | 1 | 2 | 2 | 1 | 2 | 3 | 2 | 21 |
| Voigt | 4 | 3 | 3 | 5 | 5 | 4 | 5 | 3 | 5 | 3 | 40 |
| Pearson IV | 5 | 5 | 4 | 3 | 4 | 5 | 3 | 5 | 1 | 4 | 39 |
| Pearson VII | 2 | 4 | 2 | 4 | 3 | 3 | 4 | 4 | 4 | 5 | 35 |

**Table S5.** Peak fitting shape ranking for calcite peaks FWHM according to the average errors per system.

| **Calcite** (FWHM) | S1^532^ | S2^532^ | S3^532^ | S4^532^ | S5^532^ | S6^633^ | S7^785^ | S8^785^ | S9^785^ | S10^785^ | Score Sum |
| --- | --- | --- | --- | --- | --- | --- | --- | --- | --- | --- | --- |
| Gaussian | 4 | 3 | 1 | 2 | 1 | 1 | 2 | 1 | 3 | 1 | 19 |
| Lorentzian | 1 | 4 | 4 | 1 | 2 | 2 | 1 | 4 | 2 | 2 | 23 |
| Voigt | 3 | 2 | 3 | 4 | 3 | 3 | 5 | 3 | 5 | 3 | 34 |
| Pearson IV | 5 | 5 | 5 | 5 | 5 | 5 | 3 | 5 | 1 | 5 | 44 |
| Pearson VII | 2 | 1 | 2 | 3 | 4 | 4 | 4 | 2 | 4 | 4 | 30 |

**Table S6.** Peak fitting shape ranking for polystyrene peaks position according to the average errors per system.

| **PS** (position) | S1^532^ | S2^532^ | S3^532^ | S4^532^ | S5^532^ | S6^633^ | S7^785^ | S8^785^ | S9^785^ | S10^785^ | Score Sum |
| --- | --- | --- | --- | --- | --- | --- | --- | --- | --- | --- | --- |
| Gaussian | 2 | 3 | 1 | 4 | 3 | 1 | 1 | 4 | 4 | 1 | 24 |
| Lorentzian | 3 | 2 | 2 | 2 | 2 | 2 | 2 | 2 | 2 | 2 | 21 |
| Voigt | 5 | 5 | 4 | 5 | 5 | 5 | 3 | 3 | 5 | 4 | 44 |
| Pearson IV | 1 | 1 | 5 | 1 | 1 | 3 | 5 | 1 | 3 | 3 | 24 |
| Pearson VII | 4 | 4 | 3 | 3 | 4 | 4 | 4 | 5 | 1 | 5 | 37 |

# Average error of the fit FWHM of polystyrene


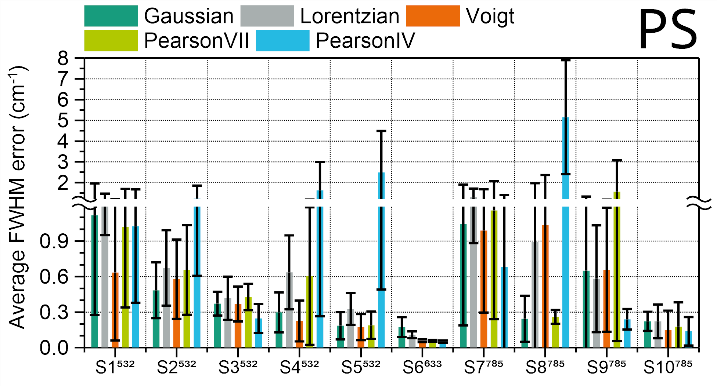


**Figure S3**. Average error of the fit FWHM of four polystyrene peaks in 10 different systems, calculated using different peak shapes. Error bars are the standard deviation.
